# Angiotensin-(1-7) Downregulates Diabetes-Induced cGMP Phosphodiesterase Activation in Rat Corpus Cavernosum

**DOI:** 10.1155/2017/5084961

**Published:** 2017-02-19

**Authors:** Gursev S. Dhaunsi, Mariam Yousif, Batoul Makki, Saghir Akhtar, Ibrahim F. Benter

**Affiliations:** ^1^Department of Pediatrics, Faculty of Medicine, Kuwait University, Jabriya, Kuwait; ^2^Department of Pharmacology and Toxicology, Faculty of Medicine, Kuwait University, Jabriya, Kuwait

## Abstract

Molecular mechanisms of the beneficial effects of angiotensin-(1-7), Ang-(1-7), in diabetes-related complications, including erectile dysfunction, remain unclear. We examined the effect of diabetes and/or Ang-(1-7) treatment on vascular reactivity and cyclic guanosine monophosphate (cGMP) phosphodiesterase (PDE) in corpus cavernosum. Male Wistar rats were grouped as (1) control, (2) diabetic (streptozotocin, STZ, treated), (3) control + Ang-(1-7), and (4) diabetic + Ang-(1-7). Following 3 weeks of Ang-(1-7) treatment subsequent to induction of diabetes, rats were sacrificed. Penile cavernosal tissue was isolated to measure vascular reactivity, PDE gene expression and activity, and levels of p38MAP kinase, nitrites, and cGMP. Carbachol-induced vasorelaxant response after preincubation of corpus cavernosum with PE was significantly attenuated in diabetic rats, and Ang-(1-7) markedly corrected the diabetes-induced impairment. Gene expression and activity of PDE and p38MAP kinase were significantly increased in cavernosal tissue of diabetic rats, and Ang-(1-7) markedly attenuated STZ-induced effects. Ang-(1-7) significantly increased the levels of nitrite and cGMP in cavernosal tissue of control and diabetic rats. Cavernosal tissue of diabetic rats had significantly reduced cGMP levels and Ang-(1-7) markedly prevented the STZ-induced cGMP depletion. This study demonstrates that attenuation of diabetes-induced PDE activity might be one of the key mechanisms in the beneficial effects of Ang-(1-7).

## 1. Introduction

Diabetes is a major risk factor for erectile dysfunction (ED). Diabetic ED is multifactorial in etiology and is more severe and more resistant to treatment compared with nondiabetic ED [1–3]. The prevalence of ED in diabetic men is estimated to be threefold higher than in the general population. Diabetes degrades both neural and vascular endothelium penile control systems [2].

Experimental evidence suggests that hyperglycemia causes elevated oxidative stress and the formation of advanced glycation and lipoxygenation end products that lead to functional loss and degenerative changes. Recent work has implicated both the renin-angiotensin-aldosterone system (RAAS) and reactive oxygen species (ROS) in the pathogenesis and vascular complications of diabetes [4, 5]. These mechanisms have effects on the regulation of nitric oxide (NO) synthase expression and activity and on proinflammatory cell signaling pathways [6]. Penile erection results from the relaxation of smooth muscle mediated by a spinal reflex to the sensory and mental stimuli. In the L-arginine–NO–guanylyl cyclase–cyclic guanosine monophosphate (cGMP) pathway, cGMP is the intracellular trigger that activates cGMP-dependent protein kinase to phosphorylate several proteins that bring about erection through relaxation of arterial and trabecular smooth muscle in corpus cavernosum due to reduced intracellular calcium levels. Since cGMP plays a key role in this process, potential interventions for inadequate smooth muscle relaxation and erectile dysfunction include elevating the intracellular cGMP levels through inhibition of phosphodiesterase (PDE) activity. PDE normally inhibits penile erection by depleting cGMP. At least more than 10 PDE families have been identified, but PDE5 is the predominant phosphodiesterase in the corpus cavernosum [7]. It has been demonstrated that treatment with PDE inhibitors such as sildenafil (Viagra) is less effective in diabetic patients for unknown reasons and gene therapy with endothelial NO synthase fails to totally restore erectile function in diabetes [8]. Furthermore these therapies do not address the underlying causes of diabetes-induced ED.

We have previously reported that older and diabetic rabbits showed impaired Ang-(1-7)-mediated relaxation suggesting that aging and diabetes-related erectile dysfunction (ED) may be partly due to decreased Ang-(1-7)-mediated relaxation of the corpus cavernosum [9]. In addition, we have also found that acute preincubation with Ang-(1-7) was effective in attenuating Ang II-induced contraction of rabbit corpus cavernosum suggesting that the possible role of Ang-(1-7) in treatment of ED should be investigated. In this study, presented in part at the 3rd World Congress on Pharmacology [10], we investigated the effect of Ang-(1-7) on PDE at the molecular level in corpus cavernosum of diabetic rats.

## 2. Materials and Methods

### 2.1. Materials

Wistar rats (male, weighing approximately 300 g) used in this study were supplied by the Animal House, Health Sciences Center, Kuwait University. Angiotensin-(1-7), norepinephrine bitartrate, and carbachol were all purchased from Sigma Chemical Co. (St Louis, USA).

### 2.2. Methods

#### 2.2.1. Animal Groups

Animal studies were conducted in accordance with the National Institutes of Health Guide for the Care and Use of Laboratory Animals (NIH Publication number 85-23, Revised 1985) as approved by Kuwait University Research Administration.

Animals included in this study were divided into the following groups (*N* = 8/group). Group 1: nondiabetic (control) animals, Group 2: diabetic animals; Group 3: control + Ang-(1-7); Group 4: diabetic + Ang-(1-7). Diabetes was induced by a single intraperitoneal injection (55 mg/kg, body weight) of streptozotocin (STZ). Rats with fasting blood glucose levels above 250 mg/dL were declared diabetic and included in the study. Ang-(1-7) was given at a dose of 576 *μ*g/kg/day after three weeks of diabetes induction and continued for the last 3 weeks prior to sacrificing the animals. Doses of the drugs used in this study were based on our previous findings in models of hypertension and diabetes [11, 12]. The rats had free access to food and water throughout the study.

The animals were sacrificed after six weeks of inducing diabetes. Rats were anesthetized with ketamine and the penis was removed en bloc after sacrificing the animals. A ventral incision was made to expose the cavernosal tissue. The corpus cavernosum was cleaned of the adjacent tissue and cut into longitudinal strips of 2 × 10 mm.

#### 2.2.2. Vascular Reactivity Studies

Strips of the corpus cavernosum were suspended longitudinally in organ bath chambers [13] containing 25 mL Krebs Henseleit (KH) solution at pH 7.4. The composition of KH solution was as follows (mM): NaCl (118.3), KCl (4.7), CaCl2 (2.5), MgSO4 (1.2), NaHCO3 (25), KH2PO4 (1.2), and glucose (11.2). The tissue bath solution was maintained at 37°C and was aerated with 95% oxygen and 5% carbon dioxide mixture. Reactivity of the isolated strips of the corpus cavernosum was determined by measurement of changes in the isometric tension to Ang-(1-7) and carbachol, using computerized automatic organ bath LSI (Power Lab/8sp AD Instruments, Pan Lab, Spain) Letica Scientific Instruments [9, 14]. The preparations were left to stabilize for 45 min with changing KH solution at 15 min intervals. A pretension of 1.0 gm was applied and the preparations were left until a stable baseline tone was obtained. The relaxant effect to the vasodilator agonist, Ang-(1-7), was determined after precontracting the tissues with phenylephrine (PE) (3 × 10^−7^ M) added to the organ baths. After obtaining a steady level of precontraction, the vasodilator effect to Ang-(1-7) (10^−12^–10^−5^ M) was tested on different preparations of the corpus cavernosum. The response to each concentration of the agonist was left to stabilize before adding the next drug concentration. The relaxant responses were expressed as percentage reduction of the tension induced by precontraction with PE. Based on the constructed dose-response curves,* E*max (the maximal percentage of relaxation attained) and ED50 (the concentration of Ang-(1-7) required to reduce the induced tone by 50) were calculated using Graph Pad Prism software. In another group of experiments, the effect of incubation with Ang-(1-7) on the responsiveness of the corpus cavernosum to carbachol was investigated. A cumulative concentration response curve was established for carbachol, as described earlier [13]. The corpus cavernosal strips were then incubated with KH solution containing Ang-(1-7) (3 × 10^−6^) for 20 min. Following the incubation period, cumulative concentration response curves for carbachol were established in the presence of Ang-(1-7).

#### 2.2.3. Assay of Cyclic GMP Phosphodiesterase (PDE) Activity

Experiments were carried out to determine the levels of phosphodiesterase in the corpus cavernosum tissues from the different groups in the study. PDE activity was measured in penile tissue homogenates using a luminescence-based kit from Promega (Madison, WI, USA). Briefly, the PDE assay was started by adding substrate (cGMP, 10 *μ*M final concentration) to the reaction mixture containing PDE buffer and a known amount of tissue homogenate. PDE-Glo termination buffer containing 3-isobutyl-1-methylxanthine (IBMX) was added to stop the reaction. Luminescence was measured using a plate-reading luminometer following addition of PDE-Glo detection solution provided in the kit. Specific activity PDE was calculated as Relative Light Units (RLU)/*μ*g of tissue protein.

#### 2.2.4. Western Blotting Studies

Western blotting for phosphorylated forms of p38 MAP kinase (p-p38 Map kinase) was performed as described previously [15]. Strips of the corpus cavernosum were cut into small pieces and homogenized in appropriate amounts of lysis buffer, protease inhibitor, and phosphatase inhibitor cocktails A and B. Lysates were centrifuged at 12000 rpm at 4°C for 15 minutes. Supernatants were then collected and protein concentration was estimated using Lowry's method. Then the samples were mixed with sample loading buffer and loaded in the 12% SDS polyacrylamide gel for electrophoresis. Separated proteins were then transferred to PVDF membrane for detection of p-p38 MAP kinase. After blocking with buffer Blotto B, membranes were incubated with the specific primary antibodies overnight, then washed with TBST, and incubated with the horseradish peroxidase-conjugated secondary antibody for 2 hours. Protein bands were detected with Super Signal chemiluminescent substrate.

#### 2.2.5. RT-PCR Detection of cGMP-PDE

Total RNA was isolated from corpus cavernosum using Qiagen kit, including an RNase-free DNase treatment to prevent coamplification of genomic DNA. The concentration of the purified RNA was determined spectrophotometrically at 260 nm and the integrity was checked by agarose gel electrophoresis in denaturing condition. The reverse transcription was performed using iScriptH cDNA synthesis kit from Bio-Rad. GAPDH house-keeping gene was used for normalization. Specific primers for PDE-5 and GAPDH used in this study had the following sequences: PDE-5: forward, 5′-TTGGAGAGCCCTTGAACATCA-3′, reverse, 5′-GTAGCCTGTAATTTGGTCAACTTCTG-3′, GAPDH: forward 5′-AAGGTCGGAGTCAACGGATTT-3′, reverse 5′-ACCAGAGTTAAAAGCAGCCCTG-3′. The transcript levels of PDE-5 and GAPDH were quantified by PCR using iTaq DNA polymerase under the following amplification conditions: 95°C for 30 sec, 58°C or 62°C for 30 sec, 68°C for 45 sec, and 40 cycles after denaturing at 95°C for 1 min. Specificity of amplification products was assessed by their size using agarose gel electrophoresis.

#### 2.2.6. Measurement of Nitrites and cGMP

Levels of nitrites, and index of NO generation, were measured in serum and cell culture supernatants using Greiss reaction assay according to the kit purchased from Calbiochem (CA). Levels of cGMP were determined using the cGMP Immunoassay Kit (R&D Systems Inc., Minneapolis, MN) following the manufacturer's instructions. The levels were normalized by total protein in each sample.

#### 2.2.7. Statistical Analysis

Sigmoidal dose-response curves were established using nonlinear regression. The concentration of the drug is shown on the *x*-axis, while the *y*-axis plots response. The *X* values are logarithm of drugs' concentrations. Data were analyzed using Graph Pad Prism software and presented as mean ± SEM of “*n*” number of experiments as described previously. The difference was considered to be significant when *p* value was less than 0.05.

## 3. Results

### 3.1. Ex Vivo Relaxation Effect of Ang-(1-7) on Corpus Cavernosum

**Figure 1:**
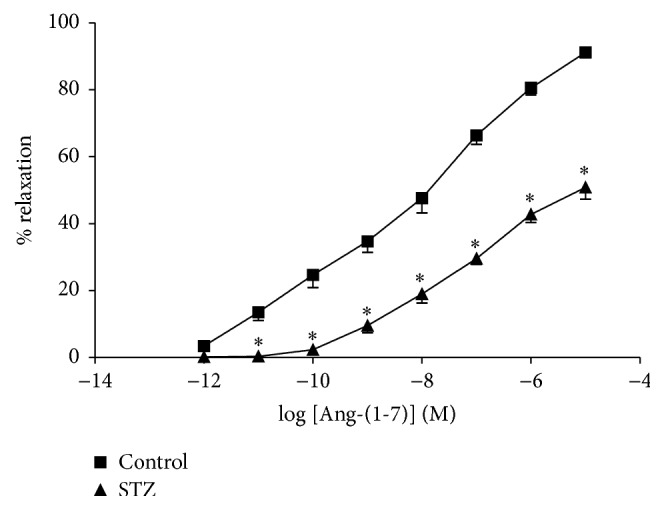
Ang-(1-7) induced relaxation (10^−12^–3 × 10^−5^ M) in the isolated corpus cavernosal strips from (■) control and (▲) STZ-induced diabetic rats. Tissues were precontracted with phenylephrine (3 × 10^−8^ M). Mean ± SEM (*n* = 5) (^*∗*^significantly different compared to control, *p* < 0.05).

**Figure 2:**
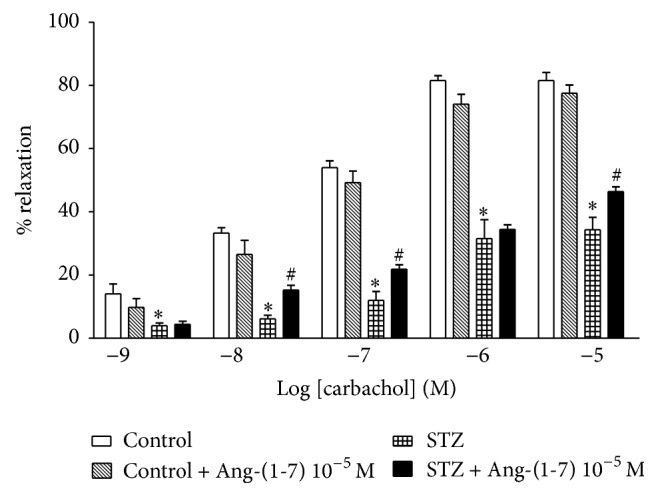
Effect of incubation with Ang-(1-7) (10^−6^ M) on carbachol-induced relaxation in the isolated corpus cavernosal strips from diabetic rats. Mean ± SEM (*n* = 5) (^*∗*^significantly different compared to control, *p* < 0.05; ^#^significantly different compared to STZ, *p* < 0.05).

Figure 1 shows that Ang-(1-7) (10^−12^–10^−5^ M) produced concentration-dependent relaxation of the corpus cavernosum isolated from control and STZ-diabetic rats. The relaxant response to Ang-(1-7) was significantly impaired in diabetic corpus cavernosum strips compared to control nondiabetic rats (Figure 1). The maximal relaxation to Ang-(1-7) was significantly reduced in the corpus cavernosum isolated from diabetic rats. The maximal percentage relaxation (*E*max) induced by Ang-(1-7) was 91 ± 2% and 51 ± 4% in the control and diabetic tissues, respectively. The ED50 values for Ang-(1-7) were significantly not different in the corpus cavernosum strips of control and diabetic rats, 7.9 ± 0.1 and 7.5 ± 0.1, respectively. The difference between the ED50 values in the two groups was not significant. Carbachol-induced relaxation of the corpus cavernosum was significantly reduced in the diabetic groups compared with the controls (Figure 2). Preincubation of the corpus cavernosum tissues with Ang-(1-7) (10^−5^ M) resulted in significant enhancement in carbachol-induced relaxant responses in the tissues isolated from diabetic rats.

### 3.2. Effect of Ang-(1-7) on Levels of Nitrites and cGMP in Corpus Cavernosum of Control and Diabetic Rats

**Figure 3:**
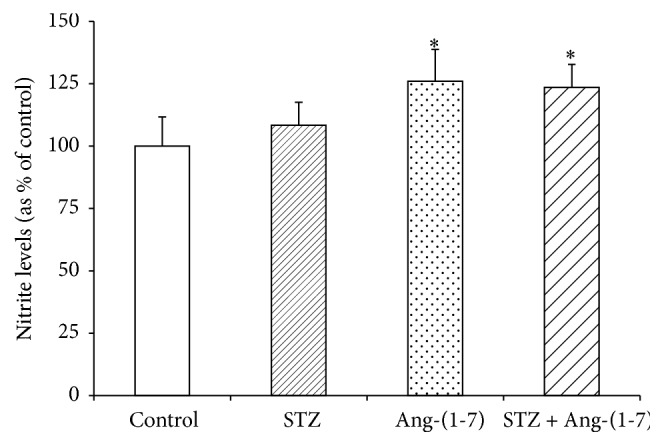
Nitrite levels in corpus cavernosum tissues from control and diabetic rats with/without Ang-(1-7) treatment. Values are mean ± SEM of six measurements and ^*∗*^*p* < 0.05 when compared with control.

**Figure 4:**
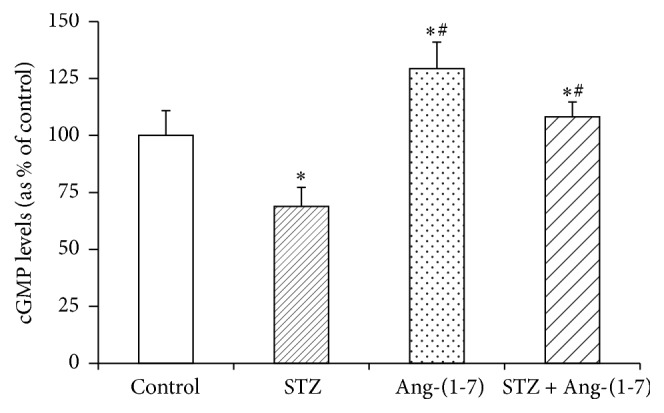
Levels of cGMP in corpus cavernosum tissues from control and diabetic rats with/without Ang-(1-7) treatment. Values are mean ± SEM of six measurements. ^*∗*^*p* < 0.05 when compared with control and ^#^*p* < 0.01 when compared with STZ-treated group.

In view of the observed relaxation effects of Ang-(1-7), levels of NO and cGMP were measured in corpus cavernosum tissue of control and diabetic rats (Figures 3 and 4). Nitrite levels in tissues from diabetic rats were not markedly different from controls; however treatment with Ang-(1-7) significantly (*p* < 0.05) increased the nitrite content in both diabetic and control tissues (Figure 3).  Figure 4 shows that cGMP levels were significantly (*p* < 0.01) decreased in corpus cavernosum of diabetic rats when compared with control tissues and Ang-(1-7) treatment significantly restored the diabetes-induced depletion of cGMP. Ang-(1-7) treatment markedly increased the cGMP levels in controls tissues also.

### 3.3. Effect of Ang-(1-7) on cGMP Phosphodiesterase in Corpus Cavernosum of Control and Diabetic Rats

**Figure 5:**
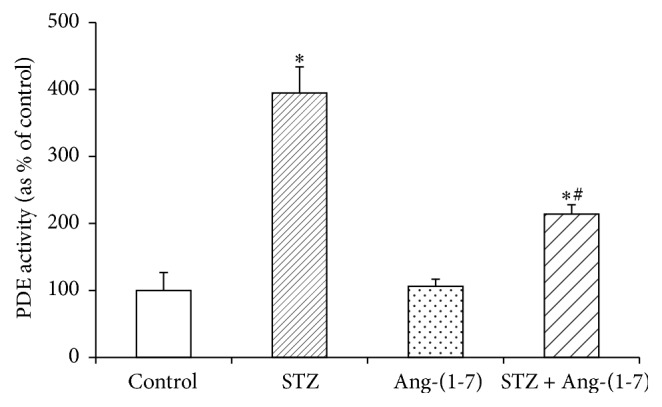
PDE levels (shown as percent of control) in corpus cavernosal tissues from control, STZ-diabetic, and Ang-(1-7) treated diabetic. Values are mean ± SEM of six measurements. ^*∗*^*p* < 0.05 when compared with control and ^#^*p* < 0.01 when compared with STZ-treated group.

**Figure 6:**
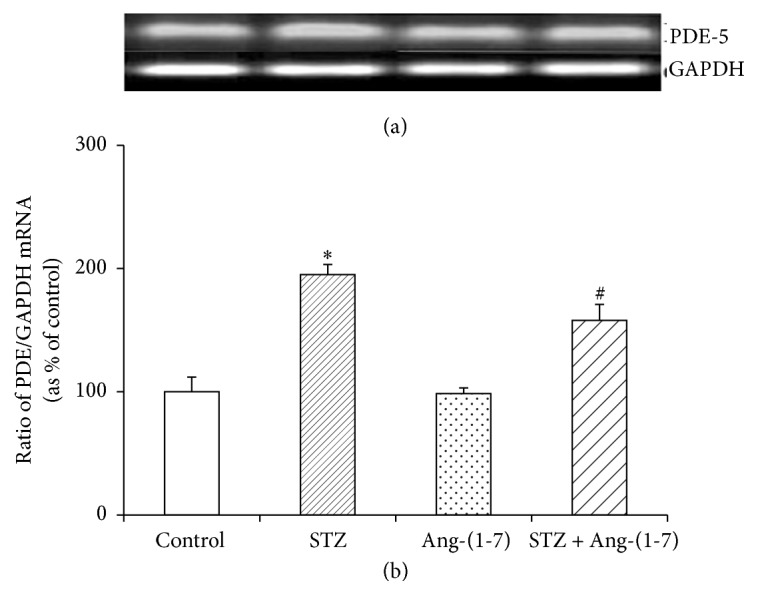
Gene expression of PDE-5 in corpus cavernosum tissues of control and diabetic rats with/without Ang-(1-7) treatment. Values (mean ± SEM of six measurements) presented as percent of control are the ratio of PDE and GAPDH mRNA. ^*∗*^*p* < 0.05 when compared with control and ^#^*p* < 0.01 when compared with STZ-treated group.

Enzymatic activity of PDE was significantly (*p* < 0.001) higher in corpus cavernosum of diabetic rats when compared with control tissues (Figure 5). Ang-(1-7) treatment did not have any marked effect on the PDE activity in control tissues but significantly (*p* < 0.01) reduced the STZ-induced increase in PDE activity. RT-PCR studies revealed that diabetes-induced enhancement of PDE activity was due to an induction of the PDE-5 gene expression (Figure 6). STZ treatment significantly increased the PDE mRNA content in corpus cavernosum and the diabetes-mediated increase in gene expression of PDE was downregulated by Ang-(1-7) treatment as evidenced by a significant decrease (*p* < 0.05) in PDE mRNA levels. Ang-(1-7), however, did not influence the gene expression of PDE in control tissues.

### 3.4. Effect of Ang-(1-7) on p38MAP Kinase in Corpus Cavernosum of Control/Diabetic Rats

**Figure 7:**
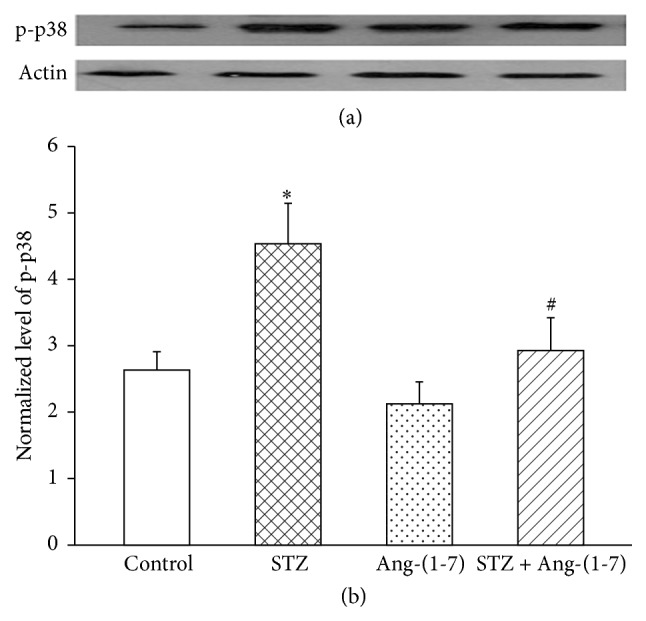
Western blot analysis of p38MAP kinase from control, control-Ang-(1-7), diabetic, and diabetic-Ang-(1-7) treated animals. (mean ± SEM, *n* = 4) (^*∗*^*p* < 0.05 compared to control, ^#^*p* < 0.05 compared to diabetic).

Western blot studies (Figure 7) revealed that protein expression of p38MAP kinase was significantly (*p* < 0.01) higher in corpus cavernosum of diabetic rats than the control tissue suggesting an association of p38MAP kinase with observed diabetes-induced increase in PDE activity. Ang-(1-7) treatment produced slight reduction in p38MAP kinase protein content of control corpus cavernosum but significantly (*p* < 0.05) corrected the diabetes-induced increase in protein expression of p38MAP kinase.

## 4. Discussion

Diabetes is a multifaceted metabolic disorder with an expanding list of clinical complications. Diabetes-related erectile dysfunction is a well-known effect of long term hyperglycemia and associated metabolic disturbances; however its pathogenic mechanisms have remained unclear. We reported earlier that Ang-(1-7) has beneficial effects against diabetes-induced erectile dysfunction and this study now demonstrates that Ang-(1-7) downregulates the gene expression of cGMP-PDE in corpus cavernosum of diabetic rats, thereby suggesting a new molecular mechanism related to diabetes-induced erectile dysfunction and the protective role of Ang-(1-7).

Our findings, in this study, show that acute ex vivo administration of Ang-(1-7) produces significant relaxing responses in corpus cavernosum of diabetic rats and also corrects the diabetes-induced impairment of carbachol relaxant responses. These results further support our previous, in vivo, studies on chronic treatment with Ang-(1-7) and strengthening the notion that Ang-(1-7) is a potent therapeutic tool to correct erectile dysfunction [13]. The fact that Ang-(1-7) treatment significantly increased the levels of NO, a major vasodilator known to play a key role in normal erectile function through activation of soluble guanylyl cyclase [16, 17], and cGMP in corpus cavernosum of control and STZ-treated rats in this study suggests that the observed relaxation effects of Ang-(1-7) are mediated via NO/cGMP. However, a marked decrease in the cGMP levels with unaltered NO levels in corpus cavernosum of diabetic rats suggests that protective effect of Ang-(1-7) against erectile dysfunction might not be solely NO-mediated but likely occurs through regulation of PDE. A remarkable increase in PDE enzyme activity in corpus cavernosum of diabetic rats justifies, at least in part, the depletion of diabetes-induced depletion of cGMP in this study and indicates that activation of PDE under hyperglycemic conditions may be a contributing factor for erectile dysfunction. Though Ang-(1-7) did not influence the PDE activity in control tissues, it markedly abated the diabetes-induced activation of PDE suggesting that Ang-(1-7) triggers compensatory responses to falling intracellular cGMP levels possibly through downregulation of PDE. Our findings that gene expression of PDE-5 was significantly enhanced in corpus cavernosum of diabetic rats and Ang-(1-7) markedly mitigated the STZ-induced overexpression, further indicating that Ang-(1-7)-mediated relaxation response might occur by increasing cGMP levels through transcriptional regulation of PDE gene. The fact that Ang-(1-7) treatment does not affect PDE gene expression in control tissue reaffirms our notion that cGMP depletion possibly triggers the Ang-(1-7)-mediated response of PDE activation.

Diabetes being a multifactorial disease involves a wide range of pathophysiological events that include development of stress due to overproduction of cytokines and reactive oxygen species [18–20]. It remains to be investigated if cellular oxidative stress is at all associated with findings of this study; however we have reported earlier that Ang-(1-7) attenuates diabetes-induced activation of NADPH oxidase in kidneys [21]. There are a number of reports that strongly link activation of p38MAP kinase with increased cellular oxidative stress [22, 23] and indication of an increase in p38MAP kinase protein expression in corpus cavernosum of diabetic rats, as was observed in this study, suggesting involvement of cellular oxidative stress in diabetes-related erectile dysfunction and supportive of earlier reports regarding association of hyperglycemia with cellular redox [24]. Observed effect of Ang-(1-7) on p38 kinase in control and STZ-treated tissues warrants further studies to examine possible association of p38MAP kinase with PDE regulation in corpus cavernosum. The findings reported herein provide a new insight into the possible molecular mechanisms of diabetes-induced erectile dysfunction and the beneficial effects of Ang-(1-7).
